# Subareolar Breast Abscess in a Male: A Case Report

**DOI:** 10.7759/cureus.42623

**Published:** 2023-07-28

**Authors:** Ryusei Yoshino, Nana Yoshida, Akane Ito, Nanami Ujiie, Masaki Nakatsubo, Yuki Kamikokura, Masahiro Kitada

**Affiliations:** 1 Thoracic Surgery and Breast Surgery, Asahikawa Medical University Hospital, Asahikawa, JPN; 2 Diagnostic Pathology, Asahikawa Medical University Hospital, Asahikawa, JPN

**Keywords:** nipple, breast cancer, male, sba, subareolar breast abscess

## Abstract

A subareolar breast abscess (SBA) is caused by the formation of an inflammatory abscess around the milk duct. SBAs usually occur in females, and reports of SBAs in males are very rare. This study reports the case of a 62-year-old male patient who presented with a subcutaneous nodule and diffuse erythema around the left nipple. Ultrasonography revealed a mixed lesion measuring 2.5 x 1.5 cm, mostly cystic. A computed tomography scan of the chest showed an irregular, nodular structure in the same area. Magnetic resonance imaging with contrast enhancement suggested an abscess. A needle biopsy was performed on the lesion, and results showed moderate inflammatory cell infiltration, including lymphocytes, plasma cells, neutrophils, and abscess formation, thus leading to the diagnosis of SBA. The patient did not strongly desire a surgical procedure. He was treated with the cephem antibiotic cefaclor and antipyretic analgesics. During the six-month healing period, cefaclor was administered for a total of six weeks. Once he improved, recurrence was observed two years after the onset of the disease; however, the symptoms improved with conservative treatment, such as warm compresses. Preventive measures should be considered as SBAs are prone to recurrence.

## Introduction

Diseases related to the mammary glands mostly occur in women, but breast lesions may also occur in men. Among the breast diseases that occur in men, subareolar breast abscess (SBA) is a relatively rare condition. To our knowledge, only a few case reports have been published, and therefore, information on its clinical features and treatment is lacking [1,2]. An SBA is characterized by the formation of inflammatory abscesses, mainly around the milk ducts. Similar to mammary gland diseases in women, the inflammation and infection of the mammary tissue in men can cause symptoms such as discomfort, swelling, heat, and purulent discharge around the nipple [3].

This report describes the case of a male patient with an SBA. We describe in detail the patient’s clinical presentation, imaging findings, pathological findings, and course of treatment to further our understanding of this rare condition in males. By considering previous literature reports, we may be able to improve the quality of life of male patients in the future by enabling early diagnosis and the selection of appropriate treatments.

## Case presentation

**Figure 1:**
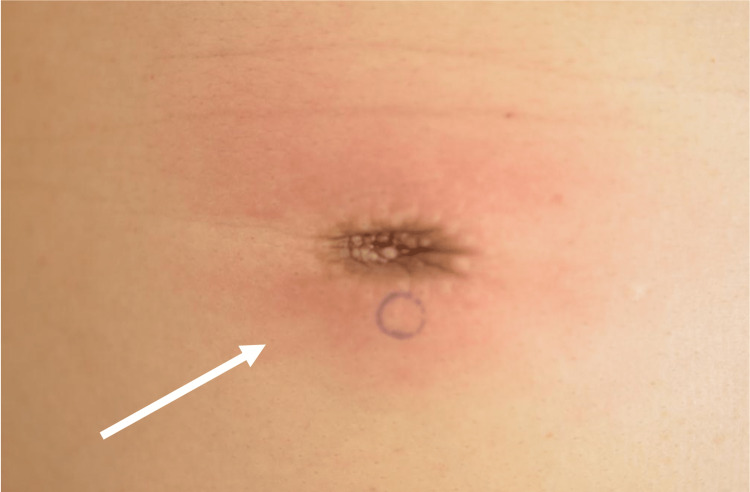
Physical examination findings Erythema diffusum was observed on the surface of the subcutaneous nodule around the left nipple with a heat sensation (arrow). These findings clearly indicated inflammation.

**Figure 2:**
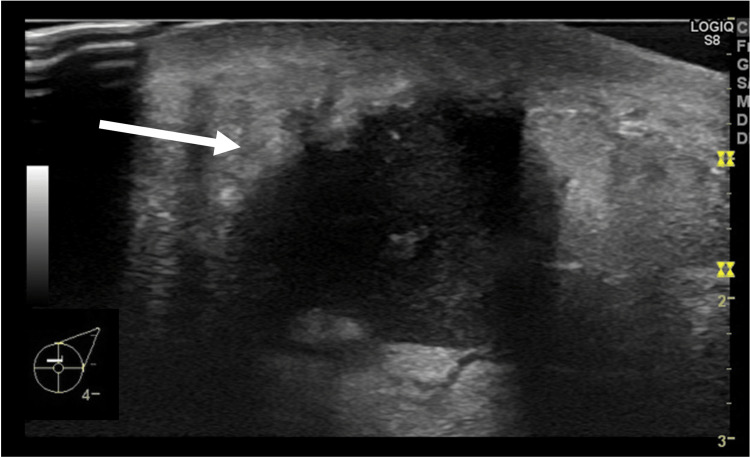
Breast ultrasound findings A 2.5 × 1.5 cm mass structure was observed. The lesions were mostly cystic, but some were solid. There was no halo, no rupture of the mammary border, and no evidence of blood flow (arrow).

**Figure 3:**
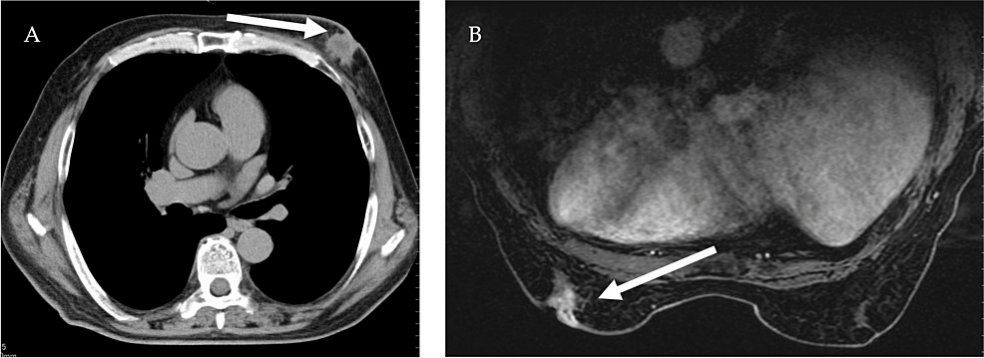
Chest CT findings and contrast-enhanced mammography MRI findings (T2-weighted imaging) (A) CT scan of the chest showed an irregular nodular structure under the left nipple with a low absorptive area inside (arrow). (B) MRI with contrast-enhanced mammography showed an irregular nodule under the left nipple with a strong enhancement effect and a marginal predominance (arrow). The enhancement effect was negative for breast cancer.

The patient was a 62-year-old male with no significant findings regarding the medical history or medications. At the age of 20, he smoked 20 cigarettes per day and drank 350 mL of beer per day, as reported. The patient visited a dermatologist because of the appearance of a subcutaneous nodule around the left nipple, which tended to increase in size. A physical examination revealed diffuse erythema and a burning sensation on the surface of the subcutaneous nodule around the left nipple (Figure 1). There were no enlarged lymph nodes in the axillary or supraclavicular areas. Ultrasonography was performed on the lesion and revealed a mixed lesion measuring 2.5 x 1.5 cm, mostly cystic (Figure 2). Computed tomography (CT) showed irregular nodular structures in the lesion (Figure 3A).

**Figure 4:**
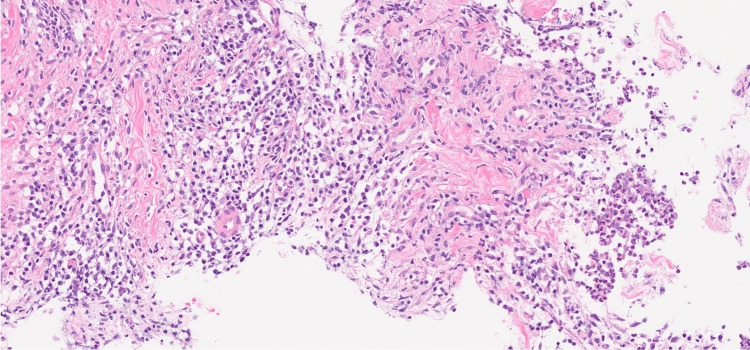
Histopathological findings Moderate inflammatory cell infiltration, including lymphocytes, plasma cells, and neutrophils, and abscess formation were observed (H&E staining, ×20).

An examination of the blood sample showed a mildly elevated white blood cell count of 9070/μL (normal 3300-8600/μL), neutrophils 70.5%, but there was no elevated inflammatory response with a C-reactive protein level of 0.18 mg/dL (normal ≤0.14 mg/dL). There were no other abnormal findings, including tumor marker levels (carcinoembryonic antigen and cancer antigen 15-3). Contrast-enhanced magnetic resonance imaging (MRI) of the breast showed an irregular nodule under the left nipple, but it was smaller than that seen on the previous CT scan. Furthermore, the enhancing effect suggested that it was an abscess rather than breast cancer (Figure 3B). A needle biopsy was performed on the lesion and revealed moderate inflammatory cell infiltrates, including lymphocytes, plasma cells, neutrophils, and abscess formation. However, there were no neoplastic changes or malignant findings. Thus, inflammatory diseases, such as subareolar breast abscess, were considered (Figure 4). On the basis of these findings, the patient was diagnosed with SBA. The patient was offered an echo-guided incisional drainage of the abscessed lesion, which the patient refused. We also explained that complete healing may be poor without surgical treatment, but the patient preferred conservative treatment. The patient was treated with the cephem antibiotic cefaclor and antipyretic analgesics. Symptoms showed improvement over a six-month period. During the six-month healing period, cefaclor was administered for a total of six weeks. The patient was monitored every three months. No flare-up of the symptoms was observed, and patient monitoring was halted.

Two years after the symptom onset, the same symptoms were observed again. Hence, the patient was referred to a breast surgeon. The patient complained of redness, swelling, and pain around the left nipple. Ultrasonography revealed the same mass-like structure found in the previous occurrence. Even at the time of recurrence, the patient was offered a procedure such as echo-guided incision and drainage, but again, the patient refused. He was treated with frequent warm compresses at home without incision, drainage, or antibiotics. The wound broke down on its own within approximately one week of conservative treatment, and improvement was observed one month after the onset of symptoms. Subsequently, the patient was instructed to cover the nipple with an adhesive bandage or gauze to prevent abrasion and to perform warm compresses regularly. No recurrence had been observed until now.

## Discussion

Breast abscesses in women are usually classified into those occurring during the postpartum period and those occurring during the nonpartum period. Most postpartum abscesses occur in women of childbearing age and are associated with bacteria such as *Staphylococcus aureus *and *Streptococcus*. On the other hand, nonpartum abscesses are sometimes associated with trauma and chronic diseases such as diabetes and rheumatoid arthritis and are said to occur in women of all ages. SBA is diagnosed in more than 90% of nonpartum breast abscesses; however, reports of SBAs in men are very rare [1,2,4,5].

The pathophysiology of an SBA is not clearly understood, but several possibilities have been postulated. First, the ducts may be dilated by the formation of emboli from the squamous epithelium in the ducts, which may lead to bacterial invasion. Second, squamous metaplasia may be triggered by other factors, thus leading to keratinization and embolization. Third, periductal inflammation can cause this condition [6,7]. Risk factors include smoking, obesity, nipple piercing, various infections, trauma, and nipple inversion [2,6]. The patient had risk factors, among which were smoking and nipple inversion.

The clinical manifestations of SBAs include nipple retraction, erythema, swelling, and pain [3]. Although SBAs are generally unilateral, bilateral cases have also been reported [8]. Mammography, breast ultrasonography, CT, and MRI are often used for diagnosis, but it is difficult to reach a definitive diagnosis. Although a few reports have been published on this, mammographic findings have shown masses, asymmetric focal shadows, and normal findings. Ultrasonography revealed cystic lesions and heterogeneous hypoechoic masses [1]. As benign signs of the disease, CT findings have shown ovality, calcification, and heterogeneous enhancing effects, and breast MRI findings have shown a high signal intensity on precontrast T1-weighted images and a low signal intensity on precontrast fat-suppressed T1-weighted images; however, none of these are specific findings for an SBA [9]. In many reports, puncture aspiration cytology was necessary for diagnosis; however, in some cases, a needle biopsy was performed to confirm the diagnosis. In addition to malignant breast cancer, gynecomastia, lipomas, schwannomas, and melanomas are listed as differential diseases. The frequency of breast cancer in men is very low, at approximately 0.5% of all breast cancers; however, early diagnosis is important because the disease is often detected in a more advanced state in men than in women owing to delayed diagnosis, and the number of cases with treatment reports is limited [1,10].

In the present case, ultrasonography could not rule out malignant findings, and the imaging findings suggested a suspicion of infection, including an abscess, rather than breast cancer. However, a definitive diagnosis could not be made. The diagnosis of SBA was made using a needle biopsy and with consideration of the possibility of breast cancer.

The optimal treatment for SBA has not yet been determined, and various treatment options have been proposed. Most patients are initially managed with antibiotics and incisional drainage of the affected area, which are often unsuccessful procedures. *Staphylococci* are the most commonly reported causative organisms, with *Pseudomonas*, *Actinomyces*, *Salmonella*, and *Listeria* also present [8]. Therefore, cephem antibiotics, which are Gram-positive broad-spectrum antibiotics, are effective in the early stages of treatment and have been used in many cases [4]. The duration of their use is largely a matter of experience, and no definitive view has been obtained. However, some cases are resistant to treatment, such as those of methicillin-resistant *Staphylococcus aureus *(MRSA) and *Pseudomonas*; culture tests can be very useful in such cases. The fact that culture tests were not performed in this case should be regretted. We believe that culture identification of the causative organisms prior to the start of treatment is necessary, bearing in mind the possibility of treatment-resistant and unexpected organisms.

Even after healing, symptom recurrences after a few months have been reported. Therefore, surgical resection is often the treatment of choice when the disease does not respond to medical therapy [11]. In most cases, drainage is performed under echo-guided guidance, and in others, incisional drainage is used. Some reports indicate that the success rate of echo-guided drainage and antibiotics is more than 80% [1]. However, when healing is still difficult, surgical resection may be the treatment of choice. The complete excision of the involved ducts is important in surgical resection to decrease the recurrence rate; however, the difficulty in identifying them is also responsible for the high recurrence rate of this disease. Although surgical resection has been reported to have a lower recurrence rate than medical therapy, it is associated with a high risk of nipple deformity and a high patient burden. Therefore, some studies recommend repeated antibiotic administration and incisional drainage [7]. Surgical treatment was not performed in our patient because the patient did not desire surgical treatment. However, it was necessary to consider echo-guided drainage or incisional drainage at the initial presentation.

In this case, the patient initially showed improvement after antibiotic treatment and incisional drainage, but the disease recurred after the long-term follow-up, similar to other cases in the literature. The patient did not wish to undergo surgery and continued medical treatment; warm compresses in the affected area might have been useful in preventing recurrence. Periodic follow-ups are necessary. However, the clinical course of this case is worth reporting due to the lack of articles describing the use of warm compresses for the treatment of SBA and its potential for preventing recurrence.

## Conclusions

A needle biopsy was useful for diagnosing an SBA in a male patient. SBA often recurs, and surgery is sometimes considered; however, using warm compresses on the lesion may be useful for the treatment and the prevention of recurrence. Considering that reports of SBAs in males are rare and that no treatment has been established, we believe that preventive treatment should be considered while bearing in mind that SBA is prone to recurrence.
